# The impact of virtual fractional flow reserve on revascularization strategy in ST-elevation myocardial infarction with multivessel disease

**DOI:** 10.1093/ehjopen/oeaf105

**Published:** 2025-08-18

**Authors:** Ioannis Skalidis, Antoinette Neylon, Francsesca Sanguineti, Mariama Akodad, Philippe Garot

**Affiliations:** Institut Cardiovasculaire Paris-Sud, Hôpital Jacques Cartier, Ramsay-Santé, 6 Avenue du Noyer Lambert, Massy 91300, France; Institut Cardiovasculaire Paris-Sud, Hôpital Jacques Cartier, Ramsay-Santé, 6 Avenue du Noyer Lambert, Massy 91300, France; Institut Cardiovasculaire Paris-Sud, Hôpital Jacques Cartier, Ramsay-Santé, 6 Avenue du Noyer Lambert, Massy 91300, France; Institut Cardiovasculaire Paris-Sud, Hôpital Jacques Cartier, Ramsay-Santé, 6 Avenue du Noyer Lambert, Massy 91300, France; Institut Cardiovasculaire Paris-Sud, Hôpital Jacques Cartier, Ramsay-Santé, 6 Avenue du Noyer Lambert, Massy 91300, France

**Keywords:** vFFR, Angiography-derived FFR, STEMI, Non-culprit lesion

**This correspondence refers to ‘Virtual physiological analysis of non-culprit disease in patients with STEMI and multivessel disease: a substudy of the COMPLETE trial’, by G.J. Williams *et al.*  https://doi.org/10.1093/ehjopen/oeaf057**

In recent years, growing attention has been given to the optimal management of non-culprit lesions (NCLs) in patients with ST-elevation myocardial infarction (STEMI) and multivessel coronary artery disease. While complete revascularization has been shown to improve clinical outcomes compared to culprit-only strategies, the precise mechanisms underpinning these benefits remain incompletely understood. Notably, a disconnect often exists between anatomical stenosis severity and its physiological significance, as assessed by fractional flow reserve (FFR) or its angiography-derived analogues. Against this backdrop, the integration of virtual physiological assessment into clinical decision-making offers a compelling opportunity to re-evaluate the role of functional lesion significance in guiding revascularization strategies.^1,2^

The substudy of the COMPLETE trial by Williams *et al*.^3^ offers important insights into the role of virtual fractional flow reserve (vFFR) in guiding NCL management in STEMI patients with multivessel disease. This work, leveraging computational physiology on archived angiographic data, sheds light on the physiological significance of bystander lesions and the clinical implications of revascularization strategies based on angiographic vs. physiological assessment.

A particularly noteworthy finding is that over half of the analysed patients exhibited no physiologically significant lesions (vFFR > 0.80), yet complete revascularization conferred clinical benefit regardless of physiological lesion severity. This observation prompts critical reflection on the mechanistic basis of benefit in the COMPLETE trial. Could it be that the therapeutic gain stems not from alleviating ischaemia *per se*, but rather from stabilizing potentially vulnerable plaque—independent of its haemodynamic impact? This hypothesis aligns with emerging evidence that plaque composition, more than luminal narrowing, predicts adverse outcomes in acute coronary syndromes.

Second, the study illustrates the limitations of visual and conventional quantitative angiographic assessment in approximating physiological severity. The weak correlation between vFFR and visual or 2D-QCA estimates (r = −0.21 and −0.15, respectively), coupled with poor agreement by dichotomous thresholds, reiterates the known discrepancy between anatomical and functional assessments. Conversely, 3D-QCA showed a substantially stronger correlation with vFFR (r = −0.60, κ = 0.47), suggesting that advanced anatomical reconstructions may more closely reflect physiological impairment. In clinical practice, however, such tools remain underutilized, and the feasibility of widespread 3D-QCA integration merits further exploration.

Third, the lack of significant interaction between physiological lesion severity and the benefit of complete revascularization raises important questions about the optimal strategy in multivessel STEMI. Should operators rely on angiographic severity as in COMPLETE, or restrict interventions to lesions with documented physiological significance, as in COMPARE-ACUTE or DANAMI-3–PRIMULTI? FLOWER-MI, which randomized patients to FFR-guided vs. angiography-guided complete revascularization, found no superiority of FFR-guidance, yet was underpowered and limited by a shorter follow-up. In this context, the current study enriches the discourse by highlighting that physiologically non-significant lesions, when revascularized, still yielded a reduction in ischaemia-driven revascularization, and potentially in MI and cardiovascular death, albeit with wide confidence intervals.

In light of these findings, one might ask: does the operator’s subjective angiographic assessment reflect an intuitive integration of features beyond stenosis severity—such as plaque morphology or vessel remodelling—that remain unquantified but prognostically relevant? Or could the early preventive stenting of angiographically moderate but biologically vulnerable lesions explain the benefit observed with complete angiographic revascularization?

Future trials incorporating multimodal imaging (e.g. OCT or NIRS-IVUS) alongside vFFR may provide further clarity on the interplay between plaque characteristics, functional severity, and outcomes. Until then, this work underscores the nuanced reality that in the setting of STEMI and multivessel disease, physiological data alone may not fully capture the complexity of risk, and that complete revascularization—guided even by angiography—may confer benefit beyond relieving ischaemia.

## Lead author biography


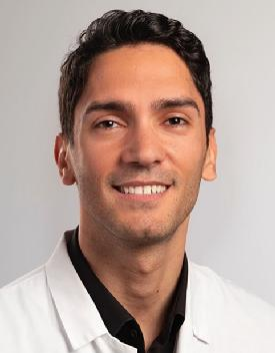


Ioannis Skalidis, MD, PhD, is an interventional cardiologist at Institut Cardiovasculaire Paris Sud (ICPS) in France. He completed his core cardiology training at the University Hospital of Lausanne (CHUV) in Switzerland and holds a PhD degree from the University of Crete. Dr Skalidis is a Swiss Board (FMH) certified cardiologist and serves as a member of the European Society of Cardiology’s e-Cardiology Working Group. His academic interests encompass coronary physiology, TAVR, and the integration of artificial intelligence and simulations in transcatheter coronary and structural heart interventions. He has authored over 100 peer-reviewed publications.

## Data Availability

No new data were generated or analysed in support of this research.

## References

[1] Skalidis I, Akodad M, Neylon A, Garot J, Sanguineti F. Angiography-derived microvascular dysfunction evaluation in patients with diabetes and ST-segment elevation myocardial infarction. Can J Cardiol 2025:S0828-282X(25)00387-3. 10.1016/j.cjca.2025.06.004 40513823

[2] Skalidis I, Noirclerc N, Meier D, Luangphiphat W, Cagnina A, Mauler-Wittwer S, Mahendiran T, De Bruyne B, Candreva A, Collet C, Sonck J, Muller O, Fournier S. Head-to-head comparison of two angiography-derived fractional flow reserve techniques in patients with high-risk acute coronary syndrome: a multicenter prospective study. Int J Cardiol 2024;399:131663. 38141730 10.1016/j.ijcard.2023.131663

[3] Williams GJ, Taylor DJ, Al Baraikan A, Haley H, Ghobrial M, Knight M, Anigboro K, Rammohan V, Gosling R, Newman T, Mills M, Hose R, Wood DA, Cairns JA, Ramasundarahettige C, Khatun R, Nguyen H, Mehta SR, Storey RF, Gunn JP, Morris PD. Virtual physiological analysis of non-culprit disease in patients with STEMI and multivessel disease: a substudy of the COMPLETE trial. Eur Heart J Open 2025;5:oeaf057. 40503340 10.1093/ehjopen/oeaf057 PMC12152305

